# MRI of pelvic endometriosis: evaluation of the mr#Enzian classification and the importance of adenomyosis subtypes

**DOI:** 10.1007/s00261-024-04359-9

**Published:** 2024-05-16

**Authors:** Antonia M. Pausch, Vivien Filleböck, Merve Benli, Isabell Witzel, Andreas M. Hötker

**Affiliations:** 1 Diagnostic and Interventional Radiology, University Hospital Zurich, Raemistrasse 100, 8091 Zürich, Switzerland; 2 Department of Gynecology, University Hospital Zurich, Zürich, Switzerland

**Keywords:** MRI, #Enzian classification, Endometriosis, Adenomyosis, Deep endometriosis

## Abstract

**Purpose:**

This study aimed to investigate the utility of the #Enzian classification in magnetic resonance imaging (MRI) for endometriosis assessment, focusing on inter-reader agreement, diagnostic accuracy, and the correlation of adenomyosis with deep endometriosis (DE).

**Methods:**

This IRB- approved retrospective single-center study included 412 women who underwent MRI evaluation for endometriosis between February 2017 and June 2022. Two experienced radiologists independently analyzed MRI images using the #Enzian classification and assessed the type of adenomyosis, if any. The surgical #Enzian classification served as the gold standard for evaluating preoperative MRI results of 45 patients. Statistical analysis was performed to assess inter-reader agreement and diagnostic accuracy.

**Results:**

Inter-reader agreement was substantial to excellent (Cohen’s kappa 0.75–0.96) for most compartments except peritoneal involvement (0.39). The preoperative MRI showed mostly substantial to excellent accuracy (0.84–0.98), sensitivity (0.62–1.00), specificity (0.87–1.00), positive (0.58–1.00) and negative predictive values (0.86–1.00) for most compartments, except for peritoneal lesions (0.36, 0.17, 1.00, 1.00, 0.26 respectively). A trend with a higher prevalence of concordant DE in women with MR features of external adenomyosis compared to those with internal adenomyosis was visible (p = 0.067).

**Conclusions:**

The mr#Enzian showed mostly high inter-reader agreement and good diagnostic accuracy for various endometriosis compartments. MRI’s role is particularly significant in the context of the current paradigm shift towards medical endometriosis treatment. The inclusion of information about the type of adenomyosis in the mr#Enzian classification could enhance diagnostic accuracy and inform treatment planning.

**Graphical Abstract:**

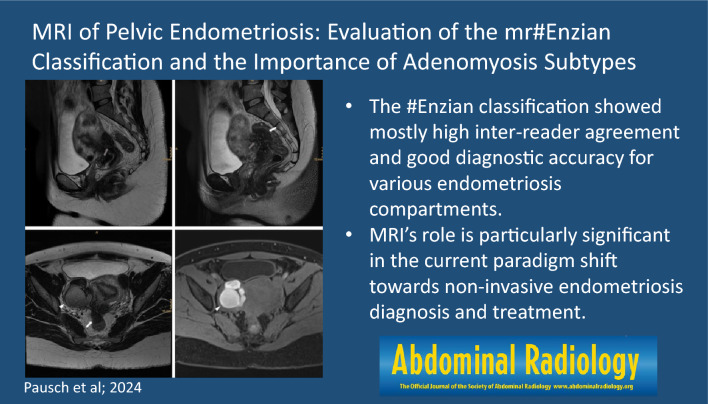

## Introduction

Endometriosis is defined as the presence of endometrial-type tissue outside the uterine cavity and, with a prevalence of approx. 10%, represents a common gynaecological disease, occurring mostly in women of reproductive age [1, 2]. Traditionally, there are three major phenotypes of endometriosis: ovarian endometrioma (OMA), superficial peritoneal endometriosis (SPE), and deep endometriosis (DE) [3]. The clinical manifestations of endometriosis, such as dysmenorrhea, dyspareunia, pelvic pain, and infertility, can vary in their presentation and complexity [4]. Consequently, diagnosing endometriosis remains challenging, often resulting in a significant delay of up to 10 years before a definitive diagnosis is made [5]. Nonetheless, timely and accurate diagnosis is crucial for effective management and improved patient outcomes. While clinical evaluation and surgical assessment have traditionally been relied upon for diagnosing endometriosis, magnetic resonance imaging (MRI) has emerged as a valuable non-invasive tool to improve detection and estimation of disease extent.

Several classification systems and scoring methods have been proposed to aid in the diagnosis and staging of endometriosis. Initially, surgical classifications, such as the revised American Society for Reproductive Medicine (rASRM) classification were established [6]. Although the rASRM classification has been adapted for MRI reporting, it does not encompass deep endometriosis [7], which may cause severe clinical symptoms and suffering. The Enzian classification [8] was primarily developed to assess deep endometriosis and is applicable in surgical, transvaginal ultrasound (TVUS), and MRI assessments. It has been recently revised by Keckstein et al. [9], now termed #Enzian classification, and includes a comprehensive evaluation of different manifestations of endometriosis, such as superficial, ovarian, deep, and extragenital endometriosis, along with pelvic adhesions [9].

This study aims to assess the utility of the #Enzian classification in MRI endometriosis evaluation, focusing on inter-reader agreement, diagnostic accuracy, and the correlation of adenomyosis with DE. Inter-reader agreement is critical for consistent and reliable diagnoses, while diagnostic accuracy has a great impact on patient management decisions and improved outcomes. In addition, understanding the correlation of adenomyosis and DE will help optimize management strategies. Ultimately, this study aims to further enhance our understanding of the utility of the #Enzian classification in MRI for endometriosis evaluation.

## Methods

In this retrospective single-center study, consecutive patients aged 18 years or older who underwent an MRI evaluation in relation to endometriosis performed at our hospital between February 2017 and June 2022 were identified. Exclusion criteria were prior hysterectomy and incomplete patient data, i.e. externally referred patient with no precise information about symptoms or medical history. The study was approved by the local ethics committee (Cantonal Ethics Committee Zurich) and informed consent was waived.

MRI scans were performed on 3.0 or 1.5 Tesla MR scanners (Siemens Skyra, Sola or Vida fit, Siemens Healthineers, Erlangen, Germany, and GE Medical Systems Discovery MR750w, GE Medical Systems, Milwaukee, WI, USA) using a dedicated pelvic MRI protocol in accordance with current guidelines [10, 11], encompassing high-resolution 2D T2-weighted TSE sequences in three orientations, 3D T1-weighted GRE sequences with and without fat-suppression and 3D T1-weighted GRE sequences with fat-suppression after contrast agent administration. In most cases, an IV anti-peristaltic agent (butylscopolamin 20 mg/ml) was injected to reduce artifacts due to bowel peristaltics before the examination. No rectal or vaginal opacification was performed.

Before the study image analysis, a training session on cases that were not part of the cohort was conducted. Subsequently, two radiologists with 5 and 3 years of experience in pelvic MRI independently reviewed the MRI images on a Picture Archiving and Communication System (PACS) workstation, while being blinded to clinical or histopathological information, except for the fact that the MRI was performed for the purpose of evaluating endometriosis.

The localization and severity grading of the endometriotic lesions and adhesions were assessed using the criteria outlined in the #Enzian classification in the publication by Keckstein et al. [9]:

For the peritoneum (P), superficial peritoneal implants with a sub-peritoneal invasion of less than 5 mm are considered. They are categorized based on the sum of all maximal diameters as: P1 < 3 cm; P2 = 3–7 cm; P3 > 7 cm.

Regarding the ovaries (O), all endometriomas and infiltrating ovarian surface foci with a size of 5 mm or larger are assessed. They are categorized based on the sum of all maximal diameters as: O1 < 3 cm; O2 = 3–7 cm; O3 > 7 cm.

The evaluation of the tubo-ovarian condition (T) involves the presence of adhesions between the ovary and pelvic sidewall with or without tubo-ovarian adhesions. The classification includes the following categories: T1 for adhesions between the ovary and pelvic sidewall, T1 plus adhesions to the uterus or isolated adhesions between the adnexa (ovaries and fallopian tubes) and uterus (T2), and T2 plus adhesions to the uterosacral ligaments (USLs) and/or bowel, or isolated adhesions between the adnexa and USLs and/or bowel (T3).

Deep endometriosis (DE) refers to implants with sub-peritoneal infiltration greater than 5 mm. The #Enzian score classifies these lesions based on their site and the involved organs. They are categorized into three compartments: Compartment A, which includes the vagina, recto-vaginal space, or retrocervical area (measured in the sagittal plane); Compartment B, encompassing the uterosacral and cardinal ligaments or pelvic sidewall (measured in the axial plane); and Compartment C, comprising the rectal wall up to 16 cm from the anal verge (measured in the sagittal plane). Lesions within each compartment are further described based on the sum of all maximal diameters: A/B/C1 < 1 cm, A/B/C2 = 1–3 cm, and A/B/C3 > 3 cm. The description of each compartment is provided separately.

For adenomyosis and other extragenital deep endometriosis, the #Enzian score includes additional categories: FA for uterine adenomyosis, defined as thickening of the myometrium-endometrium junction line greater than 12 mm; FB for bladder lesions involving the muscular layer; FU for ureteral lesions involving the muscular layer (both extrinsic and intrinsic); FI for lesions in the sigmoid colon, coecum, or ileum located above 16 cm from the anus; and F(…) for other lesions, such as those on the diaphragms, liver, or abdominal wall.

Paired organ compartments, including compartment O, T and B, were assessed, and documented separately for each side (left/right).

The #Enzian classification also encompasses additional information about the mobility of the ovaries and tubes, as well as tubal patency. However, these aspects are not evaluable on MRI, and were therefore not included in the present study. No differentiation between intrinsic or extrinsic ureteral endometriosis was made due to the small number of cases in the study population.

Additionally, as recommended by Manganaro et al. [12], the presence (SA 1) or absence (SA 0) of a sactosalpinx was evaluated separately for each side. Moreover the more experienced radiologist of the two readers noted the type of adenomyosis, following the readily applicable criteria proposed by Bazot et al. [13], who succinctly defined three types: internal adenomyosis (FA(i)), adenomyomas (FA(a)), and external adenomyosis (FA(e)). However, no further subclassifications of these types of adenomyosis were made to ensure a sufficient number of cases for each type. No correlation of the MRI findings for adenomyosis to histopathology could be established, as the specific type of adenomyosis is not routinely evaluated histopathologically.

Surgical interventions were performed by gynecologic surgeons with extensive experience in endometriosis surgery. Since MRI assessments are routinely conducted to plan the surgical intervention, the surgeons were aware of the preoperative evaluation findings. Removed endometriotic lesions were subsequently confirmed through histological examination. In cases where no endometriosis was observed during surgery, there was no assignment of a surgical #Enzian score since the condition was not encountered.

### Statistical analysis

The dataset was subjected to descriptive statistics for analysis, using the statistical software R.17 [14] for data analysis. Level of significance was set to 5%.

To assess inter-reader agreement on the #Enzian MRI classification between the two radiologists Cohen’s kappa coefficients (κ) with 95% confidence intervals were computed. Agreement values falling between 0.81 and 1.00 were considered to indicate excellent (or ‘almost perfect’) agreement, 0.61–0.80 indicated substantial agreement, 0.41–0.60 represented moderate agreement, 0.21–0.40 denoted fair agreement, and 0.01–0.20 signified slight agreement [15]. Ratings from 0 to 3 in the compartments P, O, T, A, B and C and the dichotomization with values of 0 indicating the absence and 1 indicating the presence of a sactosalpinx (SA) were used to analyze the inter-reader agreement. For the compartment FA ratings were treated as binary, without taking size into consideration. The compartment-specific inter-reader agreement analysis excluded #Enzian locations FB, FI, FU, and F(…) due to the low number of MR-positive findings. Inter-group comparisons between the different types of adenomyosis were performed using Fisher’s exact test excluding patients with two different types of adenomyosis.

The surgical #Enzian classification served as the gold standard for evaluating the preoperative MRI #Enzian classification. For each compartment, measures such as accuracy (ACC), agreement (Cohen’s kappa coefficient, κ), sensitivity (SENS), specificity (SPEC), positive predictive value (PPV), and negative predictive value (NPV) were computed. This analysis only evaluated patients from the study population who had complete surgical #Enzian scores, implemented since January 2021. Due to the relatively small number of patients in the cohort subset, all ratings were treated as binary. Moreover, the analysis excluded #Enzian locations FB, FI, FU and F(…) due to the low number of MR-positive findings.

## Results

**Fig. 1:**
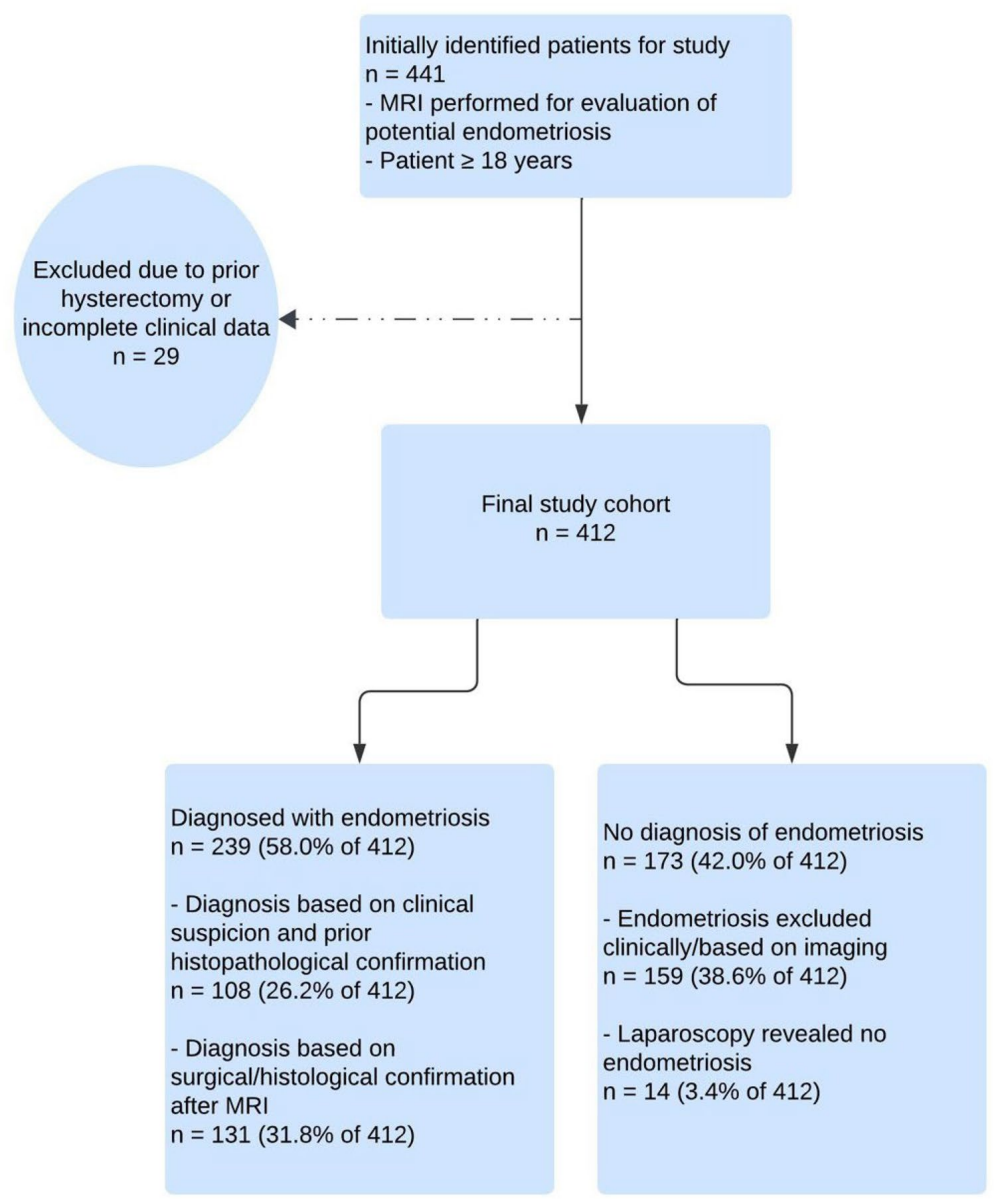
Flowchart of study cohort identification

A total of 441 women were initially identified for the study. After considering the exclusion criteria, 29 patients were excluded due to prior hysterectomy or incomplete clinical data, resulting in a final patient cohort of 412 women. In a total of 239 out of 412 patients (58.0%), endometriosis was diagnosed due to a strong clinical suspicion based on the gynecological examination and imaging in the present case along with prior surgical/histological confirmation (n = 108, 26.2%) or through surgical/histological confirmation (n = 131, 31.8%) after the study-relevant MRI examination. In 14 patients (3.4%) laparoscopy revealed no evidence for endometriosis, while in the remaining 159 patients (38.6%) endometriosis was excluded based on clinical and imaging assessments. Figure 1 depicts a flowchart detailing the study cohort identification process. Patient characteristics are summarized in Table 1.

**Table 1:** Overview of patient characteristics depicting demographics and symptoms

Patient characteristics, n = 412	
Age (years), mean ± SD	33.2 ± 7.8
Body mass index (kg/m^2^), mean ± SD	23.7 ± 4.7
*Symptoms*	
Pelvic pain, n (%)	192 (46.6)
Dysmenorrhea, n (%)	246 (59.7)
Dyspareunia, n (%)	137 (33.3)
Dyschezia, n (%)	90 (21.8)
Dysuria, n (%)	16 (3.9)
Infertility, n (%)	56 (13.6)

### Inter-reader agreement on mr#Enzian

Overall, inter-reader agreement was excellent (κ = 0.81–1.00) for mr#Enzian assessment of the ovaries, the tubo-ovarian condition, compartment A and compartment FA. Interreader-agreement was substantial (κ = 0.61–0.80) for compartment B and compartment C, and only fair (κ = 0.21–0.40) for peritoneal involvement.

In 145 out of 239 women with endometriosis (60.67%) perfect agreement was reached between the two readers with all compartments being assigned the exact same score in the MRI classifications of both readers.

**Fig. 2:**
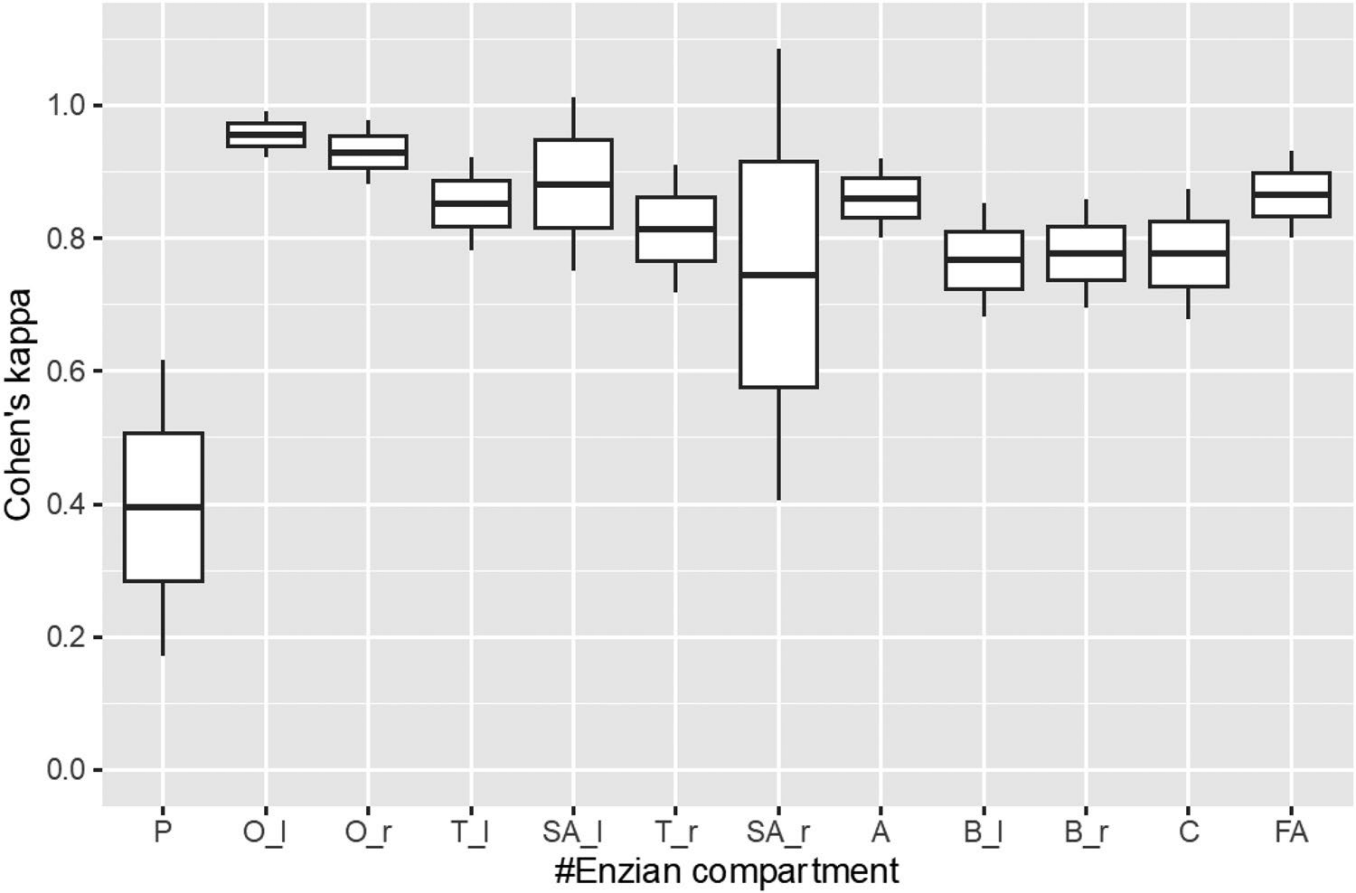
Boxplot of Cohen’s kappa values (κ) with 95% confidence intervals for inter-reader agreement as depicted in Table 2

Cohen’s Kappa coefficients for each compartment, along with their corresponding 95% confidence intervals, are presented in Table 2 and Fig. 2.

**Table 2:** Cohen’s kappa values (κ) with 95% confidence intervals (95% CI) for inter-reader agreement of the analyzed #Enzian compartments

#Enzian compartment	Cohen’s kappa (κ) 95% CI
P	0.39 (0.17–0.62)
O_left	0.96 (0.92–0.99)
O_right	0.93 (0.88–0.98)
T_left	0.85 (0.78–0.92)
SA_left	0.88 (0.75–1.01)
T_right	0.81 (0.72–0.91)
SA_right	0.75 (0.41–1.09)
A	0.86 (0.80–0.92)
B_left	0.77 (0.68–0.85)
B_right	0.78 (0.70–0.86)
C	0.78 (0.68–0.87)
FA	0.87 (0.80–0.93)

*P* peritoneum, *O* ovary, *T* tubal-ovarian condition, *SA* sactosalpinx, *A* vagina and rectovaginal, *B* sacrouterine/cardinal ligaments, pelvic sidewall, ***C*** rectum, *FA* adenomyosis

Regarding the lower prevalence compartments FB, FI, FU, and F(…), both readers reached a consensus on the detection of endometriosis as follows: FB (4 cases), FI (5 cases), FU (2 cases), and F(…) (2 cases). Due to their limited occurrence, these compartments were excluded from detailed statistical analysis, as previously outlined in the methods section.

### Comparison of mr#Enzian with surgical #Enzian score

After implementation of the new #Enzian scoring system in January 2021, 45 patients of the study population received a complete surgical #Enzian assessment at our institution, validated by histopathologic confirmation. This served as a gold standard for evaluating the preoperative #Enzian score assessed by MRI. When comparing this gold standard to the #Enzian score assigned by the experienced radiologist, the ratings were found to be identical for only 11 out of the 45 women with endometriosis (24.4%). When excluding the cases with discrepant ratings for compartment P, the number of exact matches increased to 26 out of 45 women with endometriosis (57.8%). However, regarding the individual compartments, accuracy values of more than 84% could always be achieved, except for compartment P (35.6%). Table 3 presents the accuracy (ACC), Cohen’s kappa coefficients (κ), sensitivity (SENS), specificity (SPEC) and positive and negative predictive values (PPV and NPV) for the different #Enzian compartments in the patient subset.

**Table 3:** Statistical parameters of the analyzed #Enzian compartments for the evaluation of the preoperative #Enzian score assessed by MRI in comparison to the surgical #Enzian score with histopathological validation as gold standard in 45 patients

#Enzian compartment	ACC 95% CI	Cohen’s kappa	SENS	SPEC	PPV	NPV
P	0.36 (0.22–0.51)	0.08	0.17	1.00	1.00	0.26
O_left	0.98 (0.88–1.00)	0.94	0.92	1.00	1.00	0.97
O_right	0.98 (0.88–1.00)	0.92	1.00	0.97	0.88	1.00
T_left	0.89 (0.76–0.96)	0.67	1.00	0.87	0.58	1.00
T_right	0.93 (0.82–0.99)	0.69	1.00	0.93	0.57	1.00
A	0.89 (0.76–0.96)	0.71	0.90	0.89	0.69	0.97
B_left	0.84 (0.71–0.94)	0.59	0.62	0.94	0.80	0.86
B_right	0.84 (0.71–0.94)	0.67	0.81	0.86	0.76	0.89
C	0.96 (0.85–0.99)	0.83	0.86	0.97	0.86	0.97
FA	0.87 (0.73–0.95)	0.66	0.75	0.91	0.75	0.91

P peritoneum, O ovary, T tubal-ovarian condition, A vagina and rectovaginal, B sacrouterine/cardinal ligaments, pelvic sidewall, C rectum, FA adenomyosis

*ACC* accuracy, 95% *CI* 95% confidence interval, *SENS* sensitivity, *SPEC* specificity, *PPV* positive predictive value, *NPV* negative predictive value

### Evaluation of the adenomyosis type and concordant occurrence of DE

**Fig. 3:**
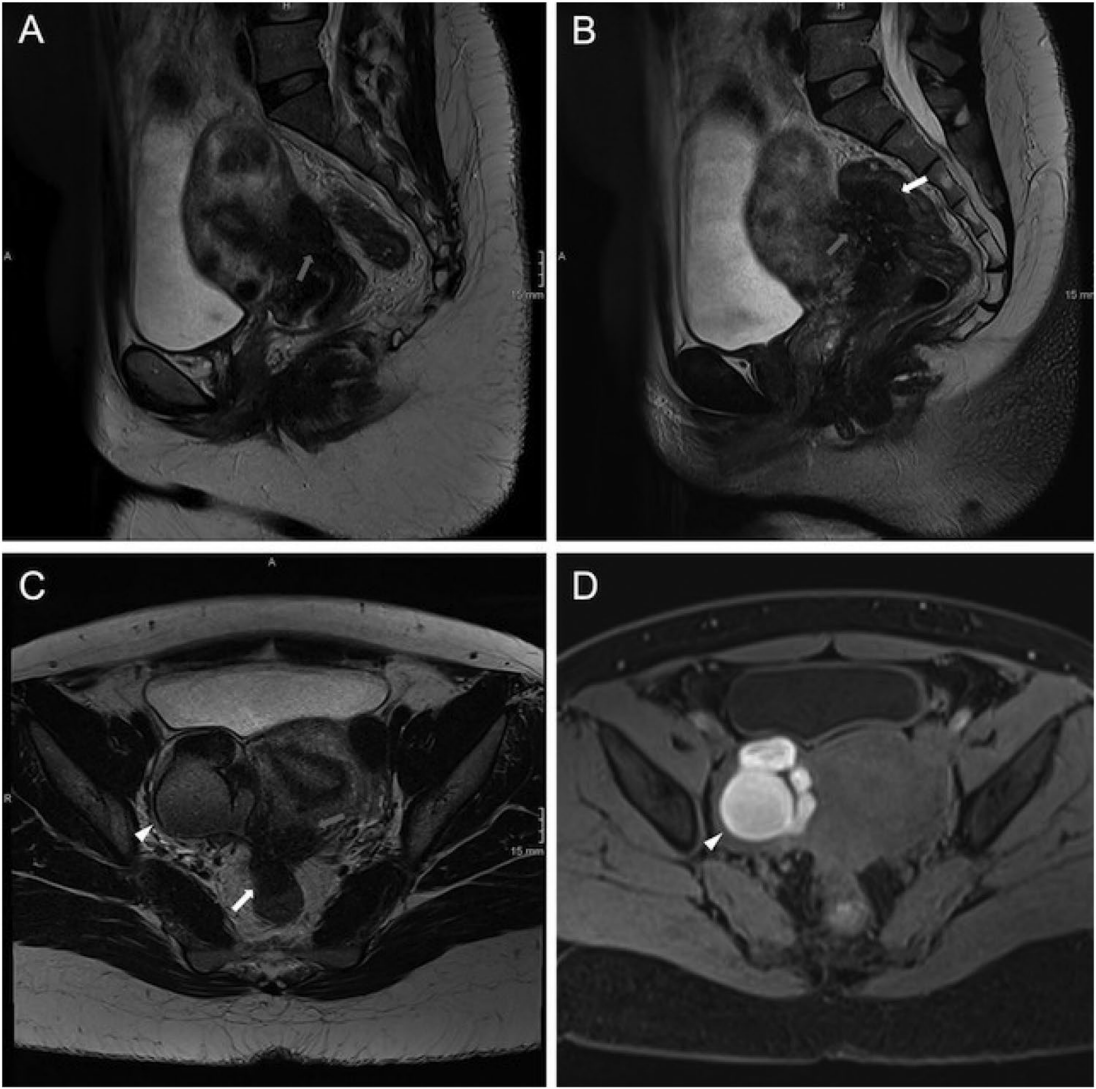
38-year-old patient with external adenomyosis of the posterior uterine wall (grey arrow in A–C) and deep endometriosis of the rectum and rectosigmoid junction (white arrow in A–C) with T2w-hyperintense foci, a T1w-hyperintense endometrioma of the right ovary (white arrowhead in C and D) with characteristic «T2-shading-sign» and adhesions between the right ovary, uterus and bowel A–C: high resolution 2D T2-weighted TSE images, D: native 3D T1-weighted GRE image with fat-suppression. mr#Enzian score: O0/2 T0/3 C3 FI FA(e)

The results of the evaluation of the type of adenomyosis are depicted in Table 4. There was a trend visible that the prevalence of deep endometriosis was higher in women with external adenomyosis compared to those with internal adenomyosis (p = 0.067). No significant difference concerning the prevalence of DE was found between women with adenomyomas and those with internal adenomyosis (p = 0.139). Figure 3 presents an example of external adenomyosis with concordant DE.

**Table 4:** Prevalence of different types of adenomyosis and concomitant deep endometriosis (DE) based on imaging

	n (%*)	Concomitant DE n (%**)
Adenomyosis	96 (40.17)	49 (51.04)
Subtypes:		
*Internal adenomyosis*	75 (31.38)	35 (46.67)
*Adenomyoma*	10 (4.18)	5 (50.00)
*External adenomyosis*	18 (7.53)	13 (72.22)
Two subtypes of adenomyosis	7 (2.92)	4 (57.14)

* Percentage in relation to the total number of patients diagnosed with endometriosis (n = 239)

** Percentage in relation to the specific adenomyosis type

## Discussion

Endometriosis poses significant diagnostic challenges, namely its heterogenous and potentially nonspecific symptomology, resulting in inadequate predictions when relying on symptom-based algorithms alone and possibly long delays in diagnosis [4]. MRI plays a crucial role in this context as it allows for accurate visualization of endometriotic lesions, pelvic structures, and their relationship to adjacent organs. Until recently, the diagnostic gold standard for endometriosis was considered to be the laparoscopic identification of endometriotic lesions, validated by histological confirmation [16]. However, laparoscopy may also have some limitations in identifying deep endometriotic lesions obscured by adhesions and inflammation, and difficulties in predicting the depth of invading rectosigmoid lesions [17]. Partly due to improved access and advances in imaging modalities, the European Society for Human Reproduction and Embryology (ESHRE) now recommends diagnostic laparoscopy only in patients with negative imaging results and/or in cases where empirical treatment was either unsuitable or unsuccessful [18]. As a paradigm shift is currently favoring empirical medical treatment, when feasible, future assessments of endometriosis will consequently rely on non-invasive methods to ensure early treatment [19].

The new #Enzian classification [9] allows for a comprehensive and structured assessment of various manifestations of endometriosis. Our study revealed a substantial to excellent inter-reader agreement for MRI assessment of all compartments except for the peritoneum with only fair agreement. This aligns with a recent study by Manganaro et al., which also demonstrated a substantial overall inter-reader agreement (κ = 0.73) and feasibility of the #Enzian score applied to MRI [12]. A trial conducted by Thomassin-Nagarra [20] addressed the inter-reader agreement between two radiologists for the MRI-based Enzian classification in 150 cases with deep endometriosis. The authors also found a substantial inter-reader agreement for compartment A (vagina, recto-vaginal space, or retrocervical area; κ = 0.79), an excellent for compartment C (rectum; κ = 0.88) and a moderate for compartment B (uterosacral and cardinal ligaments or pelvic sidewall; κ = 0.41). Furthermore, we were able to verify the inter-reader reliability of the #Enzian classification within the largest cohort to date, consisting of 412 patients.

When comparing MRI with surgical assessment, the preoperative MRI examination showed a substantial to excellent accuracy (0.84–0.98), sensitivity (0.62–1.00), specificity (0.87–1.00), positive (0.58–1.00) and negative (0.86–1.00) predictive values for detecting or ruling out endometriosis across most #Enzian compartments in our study–again, in part except for peritoneal lesions (0.36, 0.08, 0.17, 1.00, 1.00, 0.26 respectively). Despite a limited number of 45 patients included in this analysis, our results mostly align with previous studies on MRI endometriosis assessment using the earlier Enzian classification, which primarily focused on the identification of deep endometriosis, not considering compartments P, O, and T. Accordingly, a prospective multicenter study conducted by Enzelsberger et al. [21] revealed high sensitivities of up to 0.79 and specificities of up to 0.92 based on preoperative MRI findings relative to surgical findings. Similarly, in a retrospective study by Burla et al. [22] high sensitivities of up to 0.95 as well as high specificities of up to 1.00 were observed. A trial by Thomassin-Naggara et al. [20] identified in part lower accuracy values of 0.35–0.79 for MRI when compared to laparoscopy in identifying pelvic DE.

These findings collectively suggest the significant potential of MRI in detecting endometriosis across the various compartments, strengthening the argument for its application in preoperative assessment. Detecting nodules of peritoneal endometriosis with MRI is known to be challenging, especially when the lesions are small and non-hemorrhagic. However, the preoperative identification of endometriotic peritoneal implants typically does not necessarily alter the surgical approach. As a result, minor undefined peritoneal lesions could potentially be neglected [16]. Furthermore, the difficulties associated with detecting and correctly interpreting signs of adhesions in MRI, as reflected by the #Enzian compartment T, likely constitute a limitation of MRI evaluation [12, 16]. This could explain the rather low Cohen’s kappa values of 0.67–0.69 found in our study. As suggested by Manganaro et al. [12], we therefore also advocate for the evaluation of the presence or absence of a sactosalpinx and the specific fluid content (hydrosalpinx, hematosalpinx or probable pyosalpinx) to enhance the diagnostic accuracy of the tubo-ovarian condition, as already performed in our study with substantial inter-reader agreement. In addition, the relatively lower values for Cohen’s kappa and sensitivity observed in compartment B can be attributed to specific challenges associated with accurately measuring disease involvement of the uterosacral ligaments. Notably, endometriosis in this area may manifest as asymmetrical diffuse ligament thickening, which can vary significantly between patients and is often difficult to quantify with precision [16].

Moreover, our study revealed that there was a noticeable trend showing a higher prevalence of deep endometriosis in women with external adenomyosis compared to those with internal adenomyosis (p = 0.067). This result aligns with a study by Bourdon et al., who identified a significantly higher proportion of DE in the external adenomyosis-affected group compared to the internal adenomyosis-affected group [23]. One potential histopathological explanation for this might be the invasion of adjacent pelvic endometriosis into the outer myometrium [24, 25]. As recently reported in detail by Zhang et al. [26], MRI offers a precise, non-invasive tool for diagnosing adenomyosis, as the different MRI phenotypes may each be potentially linked to different causes, symptoms, and patient outcomes [23, 24, 27]. Consequently, information on the specific subtype of adenomyosis could be crucial for tailoring appropriate treatment strategies. Based on our results we therefore favor the integration of the type of adenomyosis (internal adenomyosis, adenomyomas, external adenomyosis) into the #Enzian report. However, further research is needed to fully understand and validate the clinical benefits of our study’s findings.

This study has some limitations. Firstly, the patients enrolled in this study were primarily referred by our highly subspecialized gynecology department. Consequently, there is a potential selection bias as these patients are more likely to have extensive endometriosis. Additionally, patients in the analysis of inter-reader agreement were diagnosed with endometriosis either through clinical suspicion based on gynecological examination and imaging or surgical confirmation, which also presents a potential selection bias. Furthermore, the small cohort of 45 patients with available surgical #Enzian scores and histopathology in the analysis of the diagnostic accuracy and the lack of blinding of surgeons to preoperative imaging findings could introduce bias. Lastly, the use of laparoscopy with histological confirmation as the gold standard for the second analysis, despite its known limitations, is another aspect to consider.

## Conclusion

In summary, our study further explored the role of MRI in assessing endometriosis using the #Enzian classification. We found strong agreement among readers in most compartments, indicating the reliability of MRI interpretations. Diagnostic accuracy of MRI for identifying endometriosis was mostly good, with some challenges in evaluating peritoneal lesions and the tubo-ovarian condition. The potential correlation of external adenomyosis with the presence of DE adds a new aspect to our understanding of adenomyosis phenotypes. We therefore advocate for the inclusion of information about the presence or absence of a sactosalpinx to improve the diagnostic accuracy of the tubo-ovarian condition and the subtype of adenomyosis to value the different phenotypes of adenomyosis in the mr#Enzian classification. Our findings demonstrate MRI’s potential in comprehensive endometriosis diagnosis, prompting continued exploration for enhanced accuracy and patient care.
